# Female Choice or Male Sex Drive? The Advantages of Male Body Size during Mating in *Drosophila Melanogaster*

**DOI:** 10.1371/journal.pone.0144672

**Published:** 2015-12-11

**Authors:** Santosh Jagadeeshan, Ushma Shah, Debarti Chakrabarti, Rama S. Singh

**Affiliations:** Department of Biology, McMaster University, 1280 Main Street West, Hamilton, Ontario, L8S 4K1, Canada; National Cancer Institute, UNITED STATES

## Abstract

The mating success of larger male *Drosophila melanogaster* in the laboratory and the wild has been traditionally been explained by female choice, even though the reasons are generally hard to reconcile. Female choice can explain this success by virtue of females taking less time to mate with preferred males, but so can the more aggressive or persistent courtships efforts of large males. Since mating is a negotiation between the two sexes, the behaviors of both are likely to interact and influence mating outcomes. Using a series of assays, we explored these negotiations by testing for the relative influence of male behaviors and its effect on influencing female courtship arousal threshold, which is the time taken for females to accept copulation. Our results show that large males indeed have higher copulation success compared to smaller males. Competition between two males or an increasing number of males had no influence on female sexual arousal threshold;—females therefore may have a relatively fixed ‘arousal threshold’ that must be reached before they are ready to mate, and larger males appear to be able to manipulate this threshold sooner. On the other hand, the females’ physiological and behavioral state drastically influences mating; once females have crossed the courtship arousal threshold they take less time to mate and mate indiscriminately with large and small males. Mating quicker with larger males may be misconstrued to be due to female choice; our results suggest that the mating advantage of larger males may be more a result of heightened male activity and relatively less of female choice. Body size per se may not be a trait under selection by female choice, but size likely amplifies male activity and signal outputs in courtship, allowing them to influence female arousal threshold faster.

## Introduction

Acts of mating involve interactions between two individuals, and as such, all actions associated with mating may not necessarily fulfill the dictates of natural selection, i.e. that all acts be of positive effects and good to both partners. Darwin realized that natural selection does not adequately explain the evolution of some traits, particularly traits involved in sex and reproduction [1]. He saw the need to propose a different process called Sexual Selection to make the distinction between the struggle for survival vs. struggle for the possession of mates, and recognized two processes responsible for sexual selection–male-male competition and female mate choice [1,2].

It is worth noting that much of Darwin’s explanations were largely based on the roles that males played in order to succeed in their efforts for the possession of females, and how females may respond to these efforts [1,2]. Male behaviors drew his attention to characterize two mechanisms by which males win in the struggle for reproduction: one, where the winners of male-male combat gain access to mate with females; and the other where male-male competition occurs (through a variety of displays or song) to gain the attention of the female, and females may then ‘choose’ their mates (pp 88–91 in [1]). Note that female choice may not necessarily be involved in the former. Whereas much of the sexual selection literature has dichotomized the two processes, in reality, male-male competition and female choice may best represent the extremes of a continuum of consequences involving negotiations where male-male competition and female choice interact in various ways [3,4,5,6]. It is arguably likely that the same tactics (or some of them) used by males in male-male competition may also be employed to coerce females or manipulate female choice behaviors and this can lead to sexual conflict [7]. As such, we term the abilities of males related to courtship and mating, i.e., their aggressive and/or persistent efforts to secure matings either by ‘charm’ and/or ‘coercion’, as male sex drive. This would be equivalent of what Darwin called ‘*eagerness*’ of males in order to describe the persistent efforts and strategies used by males to succeed in mating [2].

There is a need to re-examine the relative importance of female choice/preference and male eagerness (male sex drive), as well as their interactions i.e., how male behaviors elicit choice behaviors in females [3,5,6,8]. Male-male competition is more or less clear in most instances but female ‘choice’ can be quite elusive to determine experimentally. For instance, do females actively choose male victors of competition [5]? And if so, what is the basis of this choice, i.e., what character is being chosen? On the other hand, do females merely accept the male victors as long as they court females? In either case, a positive correlation between a male trait and mating success can be derived, despite evidence that females do not always choose dominant males, and mating with dominant males can sometimes be detrimental to females (see [4,6] for discussions). The underlying thinking behind the choice of the dominant or most ‘elegant’ male is that the male may represent superior quality–genetically, phenotypically or socially (as resource providers and parental caregivers) [8,9]. But we know that in many cases, males manipulate females [7], and so the best manipulators of female acceptance behaviors can also gain mating advantage. We investigate this possibility in *Drosophila*.

Male body size in *Drosophila* is a rather robust indicator of fitness as measured by mating success in a variety of species, in nature as well as in the laboratory [10,11,12,13,14,15]. Larger males have been reported to out-compete smaller males in aggression, court more vigorously and spend less time courting to achieve copulation [10,13,14,16,17,18]. The only indication of any form of female choice of larger males is the suggested possibility that females may somehow respond better to larger males who are ‘better, more audible’ singers [13], perhaps as a result of having larger wings [11,12]. Other studies have used the mating advantages of larger males as (indirect) predictors of being preferred by females. For instance, Pitnick [19] showed that females initially mated to small males, re-mated more quickly and more often with larger males. The reverse was not true, and it was surmised that this preference indicates cryptic female choice [19]. Subsequent studies testing sexual conflict have used male size as a proxy to show that mating with preferred males was detrimental to females [20,21]. Determining female choice is not only tricky in *Drosophila* [10,13,22], but there exists substantial variation in the correlation between male size and mating success within and between species [16,23]. This causes some confusion in cause and effect in sexual selection studies because body size may not be characteristic of a sexually selected trait in the traditional sense, i.e. explained by male-male competition or by female choice alone [11] but rather by some interaction of the two. For instance, body size may only serve to amplify courtship signals or male activity in mating, rather than being a preferred trait by itself. Increased signal quality would be expected to be better received by females [3].

The goal of this study was to shed light on the nature of negotiation between male mating behavior and female acceptance behavior during courtship using laboratory strains of *Drosophila melanogaster*. More specifically we wanted to examine the relative importance of male mating behaviors and female choice in influencing the mating success of males of different body sizes. This study employs a series of assays including competitive assays and assays to test the courtship threshold, which we employ as ‘courtship arousal threshold’. But rather than using re-mating propensity as a proxy for female preference [19], we use copulation interruption, immediately followed by a choice to re-copulate; the idea being to test the parameters of female arousal threshold in an effort to measure how important is female choice alone, in influencing male copulatory success. Our results show that larger males are indeed better at eliciting faster acceptance behaviors in females. Increasing male competition has little effect on mating negotiations. A novel and interesting result shows that once females have crossed the courtship arousal threshold, the relationship between male body size and mating speed and success no longer holds, i.e., females indiscriminately mate more quickly with large or small males. These results have important bearing on our understanding and the relative importance of male mating behaviors and female choice in sexual selection. The results also support Darwin’s idea of the selective advantage of male mating behaviors in driving sexual selection.

## Materials and Methods

### Fly stocks, handling, size manipulation and sex identification

Flies used in this study were obtained from a previously outbred strain established by crossing 6 different geographical strains of *D*. *melanogaster* [24]. All flies were raised on standard yeast-cornmeal and molasses medium at 25^°^C. Two culture bottles of high density population (300–400 flies) and low density population (50–150 flies) were created for size manipulation. Flies grown under crowded conditions (high density) are generally smaller in size [25]. Sexing was done at the pupal stage (~120–124 hours of development). Under a light microscope (10x), male pupae were identified as having two dark spots on the posterior-ventral region that correspond to male sex-combs. Pupae were carefully scooped up using a wet paint brush, and male and female pupae were transferred into separate vials containing culture media. Emerging adults were aged to 4 days. Prior to each experiment, individual male and female flies were anesthetized using CO_2_, and their sizes were determined. Size was determined as the length from the head to the thorax (see [20]). Males measuring 2.0–2.8 mm from the low density population were considered large, and males from the high density population measuring 1.2–1.6 mm were considered small. Females in all experiments were 2.2–2.5 mm, categorized as ‘intermediate size’. Flies not meeting these size ranges were discarded. At the time of experimentation, all flies were 5 days old and virgins. If flies were injured while transferring, the replicate was discontinued and discarded.

### Courtship arousal threshold

Courtship arousal threshold was defined as the time taken from when the male aligns himself next to the female and begins wing vibrations [26] until the time that he mounts the female and copulation begins. If courtship did not begin within 10 minutes, the replicates were discarded. If courtship occurred, but males were not able to copulate with the female within 10 minutes, the trial was termed unsuccessful. Accordingly, successful copulations reflect males who courted and copulated with females within 10 minutes. In competitive experiments involving two males, one of the males of known size were marked with a wing-clip. Experiments were conducted in 5ml beakers.

#### 1.Influence of male size on female courtship arousal threshold

This set of assays had two objectives; a) to determine how male body size (Large–L, or Small-S) influenced courtship arousal threshold (CAT) of females (F), and b) to determine if and how CAT is influenced when the female is presented with more than one male. Here we expected CAT to increase because the additional males may be a cause of distraction for males, females, or both.

1a) The first experiment involved introducing one large male to one female (L: F), or, one small male to one female (S: F). 1b). The second experiment involved introducing two males of similar sizes (LL: F or SS: F) to one female. This experiment was done in two ways: (i) In one treatment, we presented the female with a mating male (S or L)–this male was allowed to court and mate with the female. The second similarly sized male (S or L) was constrained behind a transparent, perforated barrier. The barrier was a thin, transparent plastic film. A surgical needle was used to perforate the entire barrier evenly. The transparent barrier removes physical contact between the experimental male and female, and the competitor male behind the barrier, but (presumably) allows visual perception and also allows auditory and chemical information across the barrier. These assays are presented as (S|S: F, and, L|L: F), where ‘|’ represents the barrier. (ii) In the next treatment, the size of the male behind the barrier was switched, such that treatments represented (L|S: F, and, S|L: F). 1c). The third experiment involved introducing two males of different sizes to one female (LS: F) without any barriers separating the flies.

Note that in experiments 1a to 1c, flies were anesthetized and immediately introduced into the experimental vials, where they recuperated from anesthesia.

#### 2.Effect of increasing number of males on courtship arousal threshold

In this experiment we intended to test if increasing male density alters CAT. Here flies were anesthetized, and sexes were maintained in separate vials to recuperate for 24 hours before experimentation. We exposed one female to 2, 3, 4 and 5 larger (L) or (S) males in separate experiments. The assumption here is that increasing males may represent increased male-male competition, and/or it may also represent a scenario of increasing number of choices for females. We expected that if female choice is operational, then she may take longer time to assess all males, accordingly delaying the time she might take to copulate with any male.

#### 3.Courtship interruption assays to test for female choice

The objective of this series of experiments was to test if females exercise choice after they are ‘sexually aroused’. The assumption here is that since females have ‘accepted’ to mate with a male (L or S) that has been courting her; if the female is indeed choosy and prefers larger males, then, when copulation is interrupted and the female is given a choice of the same male (L or S) and a second male (L or S), she would consistently re-copulate with the larger male. In all these assays, flies were anesthetized, and sexes were maintained in separate vials to recuperate for 24 hours before experimentation.

3a). In the first experiment, we introduced one small male to one female (S: F). If courtship did not occur within 10 minutes, the replicate was discarded. If courtship occurred, but males were not able to copulate with the female, the trial was termed unsuccessful. Accordingly, successful copulations involve males who courted and copulated with females within these 10 minutes. Once male have succeeded in mounting and copulation began, we allowed copulation for 15 seconds, and then interrupted copulation by gently shaking the vial. Immediately after interrupting copulation, a second large male (L) marked with a wing clip was introduced into the vial. If copulation did not occur within the next 10 minutes, the replicate was discarded. Successful and unsuccessful copulations were recorded. If copulation occurred, we noted which male courted and subsequently copulated with the female. Observations were made through a magnifying glass to keep track of male identify and activity.

3b). In the second experiment, we introduced one large male (L) and one female into a vial (L: F). If courtship did not occur within 10 minutes, the replicate was discarded. If courtship occurred, but males were not able to copulate with the female, the trial was termed unsuccessful. Accordingly, successful copulations reflect males who courted and copulated with females within 10 minutes. Once the male mounted and began to copulate with the female, copulation was interrupted after 15 seconds by gently shaking the vial. A second small male (S) marked with a wing clip was introduced into the vial. If copulation did not occur within 10 minutes, the replicate was discarded. Successful and unsuccessful copulations were recorded. If copulation occurred, we noted which male courted and succeeded to copulate with the female. Observations were made though a magnifying glass to track the activity of marked and unmarked males.

All statistical tests were done using Minitab 17 (Minitab Inc. 2010). The number of replicates done in each assay is indicated in the figures and/or results table.

## Results

### Influence of male size on female courtship arousal threshold

**Fig 1:**
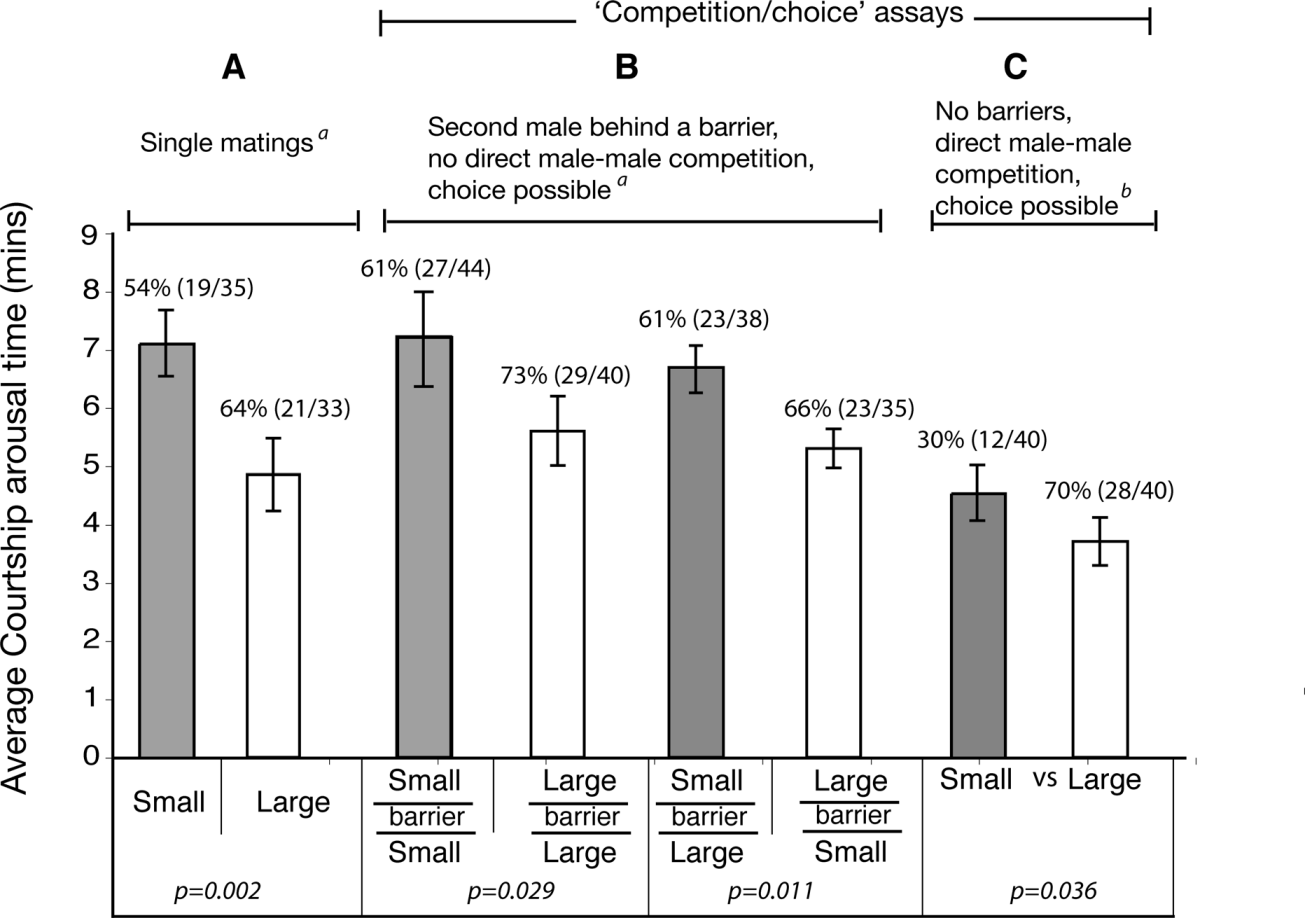
Influence of male size and ‘female choice/male competition’ on courtship arousal threshold times. (**A)**. Single matings. (**B)**. ‘Choice/competition’ assays where the second male was constrained behind a barrier. (**C)**. ‘Choice/competition’ assays without any barriers. Values above bars are percentage of successful copulations (successful copulations/trials). P-values from Mann-Whitney-Wilcoxon’s test. SE. Standard Error. ^a, b^. Time differences between ^a^ and ^b^ are due to differences in recuperation times after anesthesia (see method for details).

To determine the relationship between male body size and female courtship arousal threshold (CAT), we implemented two sets of assays. In the first experiment, a single male (large—L, or small—S) was mated with a single female (F). As expected from previous studies (see introduction), courtship arousal threshold times were shorter when females were mated singly with larger males (CAT = = L: F > S: F, p < 0.005, Fig 1A, S1 Table). We then introduced ‘female choice/male competition’ by presenting females with a second male using two different assays.

In one assay, the second male was constrained behind a transparent, perforated barrier, preventing any direct contact with the experimental male and female who were allowed to interact. We intended to test if the presence of another male (perceived by visual/auditory/chemical cues, without direct male contact/competition) changes courtship negotiation in any way. We found no evidence to support that the presence of a second male of any size behind a barrier (L|L: F, S|S: F, S|L: F, L|S: F) had an impact on CAT; i.e., CAT remained the same as in the first experiment–larger males took less time to court and copulate with females (CAT = = L > S, p < 0.05), (Fig 1B, S2 Table).

In the second ‘female choice/male competition’ assay, two males of different sizes were presented to females without any barriers (LS: F). As such, direct male-male competition as well as any female ‘assessment’ of both males was possible. In these experiments, larger males took less time to arouse and copulate with females compared to smaller males (p < 0.05, Fig 1C, S3 Table). We did not compare these results to the previous experiments (using a barrier) due to methodological differences in time allowed for flies to recuperate after anesthesia (see methods) that may affect CAT outcomes.

**Fig 2:**
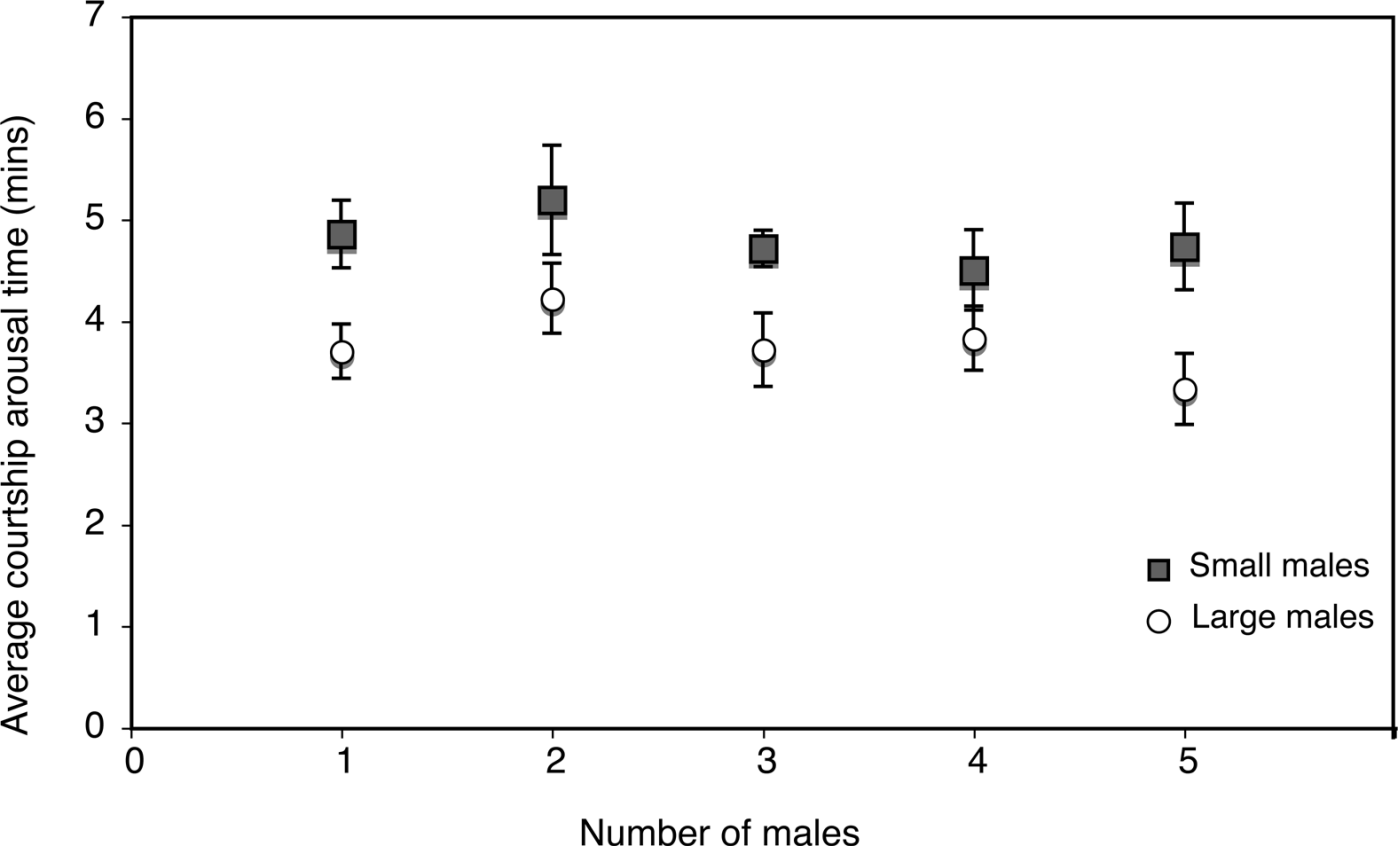
Influence of male size and increasing ‘male competition/female choice’ on courtship arousal threshold times.

We conducted another assay to test for the effect of ‘female choice/male competition’ on courtship negotiations. Here, we assessed the effect of increasing intensities of ‘female choice/male competition’ on CAT, by exposing single females to increasing number of males (1, 2, 3, 4 and 5 males of similar sizes). In these assays, we found no significant effect of increasing ‘female choice/male competition’ on CAT amongst small or amongst large males (Kruskal-Wallis; p > 0.5, Fig 2, S4 Table). However, comparing between the two, CATs in the case of large males, as expected, was significantly smaller than CATs in the case of small males (p < 0.05 in all cases).

Overall, these experiments quite clearly show that in both singly mated, and in ‘choice/competition’ matings, larger males generally took less time to arouse and copulate with females, compared to small males.

### Courtship interruption assays to test for female choice

**Fig 3:**
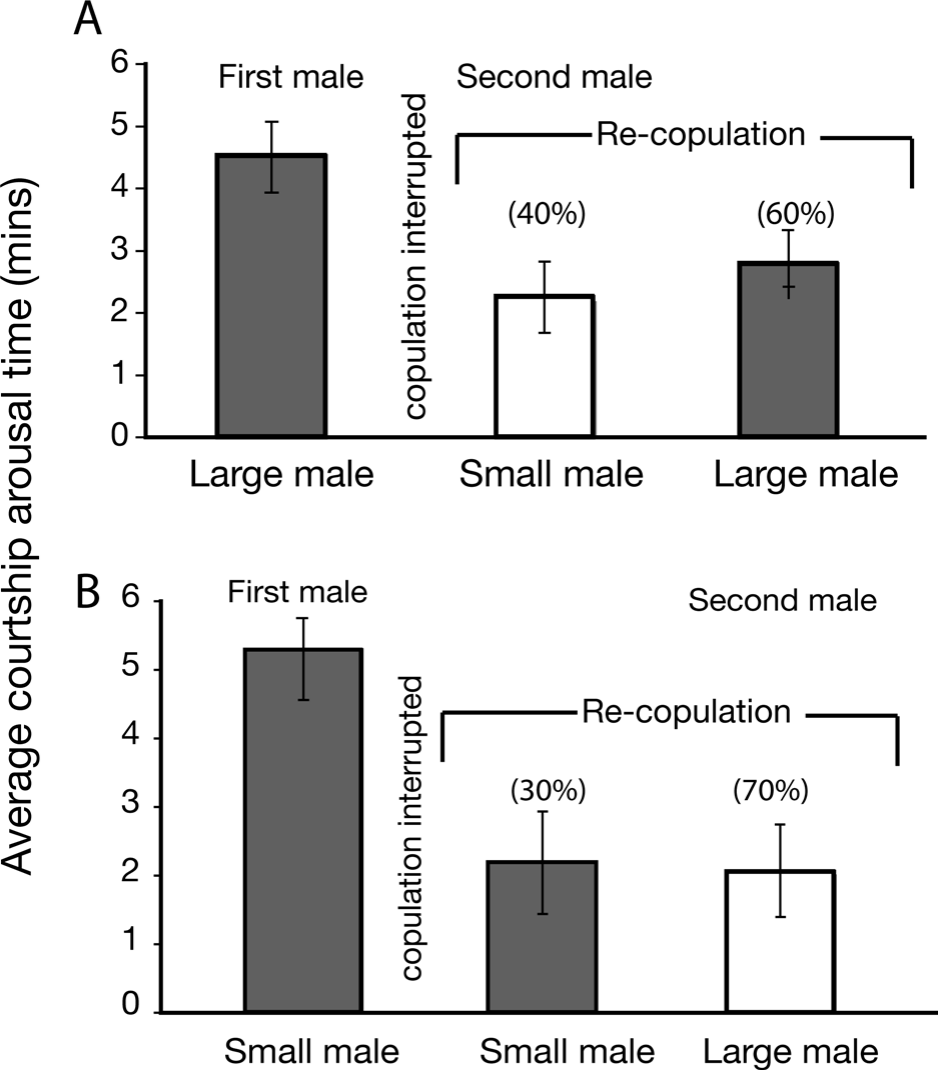
Copulation interruption assays to test for female preference between large and small males. (**A)**. First male was large, and the second male was small. (**B)**. First male was small and second male was large. Values in parentheses are percentage of successful copulations.

Courtship interruption assays were designed to test if females exercised ‘choice/preference’ after they have crossed the courtship arousal threshold. Here females were presented with a male (L, or S), and when the male began to copulate with the female, we interrupted copulation, and immediately introduced a second male of a different size (see methods for details). When the first male to copulate with females was large, copulation was interrupted, and a second small male was introduced, we found no evidence that females preferred the initial mate (L), or the second mate (S) with respect to CATs, but larger males secured more copulations (Table 1, Fig 3A, S5 Table). When the first male to copulate with the female was small–and a large male was introduced after interrupting copulation, we found no evidence to support that females preferred their initial mates (S), or mates introduced later (L) with respect to CATs but as in the previous experiment larger males secured more copulations (Table 1, Fig 3B, S5 Table).

**Table 1:** Copulation interruption assays to test for female choice.

First male	CAT1	Second male after copulation interruption	No successful re-copulations		CAT2 (re-copulation)	p-value CAT1 vs. CAT2	p-value CAT2(S) vs. CAT2(L)
Small	5.33(0.72)		Small	9/30 (30%)	2.11 (0.32)	0.0162	0.015
		Large	Large	21/30 (70%)	1.90 (0.27)	0.0257	
Large	4.53(0.66)		Large	18/30 (60%)	2.72 (0.61)	0.0279	0.020
		Small	Small	12/30 (40%)	2.42 (0.39)	0.0399	

CAT–courtship arousal threshold. p- values are from Mann-Whitney-Wilcoxon’s tests.

When we looked at CATs before copulation interruption, and after copulation interruption, even though larger first-males were able to court and mount females quicker before copulation interruption, females showed little discrimination in terms of the time they took to re-copulate with large and small males (Fig 3).

Quite interestingly, when the CATs from these experiments are compared to the previous experiments (pre-arousal) that did not involve copulation interruptions ((LS: F) where flies were treated similarly, i.e., in both cases flies were allowed to recuperate for a day after anaesthetization) the CATs after interruption (post-arousal) was significantly shorter for both small and large second-males (Table 1). Sexually aroused females re-mated quickly with any male (L or S) who courted.

## Discussion

The copulatory success of larger males, despite being attributed to female preference has generally been difficult to reconcile [16]. It may be viewed either, as females taking less time to mate with larger males (short CAT), or, (without female choice) as larger males having faster mating speeds in making females ready to mate sooner. These two views basically represent two sides of the same coin since mating is a negotiation, and our results shed light on the nature of this negotiation between the sexes. As our results show, larger males do seem to have a mating advantage by being able to court and copulate with females quicker (a short CAT) (Fig 1 and Fig 2). Competition had no influence on CAT amongst large or amongst small males (Fig 2). On the other hand, CAT is dramatically influenced by the female’s physiological and behavioral state as seen from the copulation interruption assays- females indiscriminately mate quickly with either sized male after copulation is interrupted (Table 1, Fig 3).

One explanation for the faster mating speeds of larger males compared to smaller males may be that larger males are perhaps preferred by females [19]. But another explanation is that larger males are more persistent and aggressive than smaller males in their courtship and pursuits of females [13] that they manage to court acceptance behaviors from females in a shorter period of courtship time. In competitive settings our results show that the presence of another male has little impact on CAT (Fig 1). We would note here that the assays with a second male behind a barrier were intended to test the effect of the presence of a second male (no direct combat). Our assumption was that even if visual cues are not perceived, volatile pheromones may be used to detect the presence of other males, but this may not be the case and in reality, sense by contact may be more meaningful [27]. Nevertheless, results between assays with and without a barrier are consistent in showing the copulatory success of larger males, and are consistent with observation in the laboratory as well as in nature [10,13,19]. Moreover, even an increasing number of males (a proxy for increasing ‘competition/choice’) had no significant effect on CAT amongst large or small males (Fig 2). We are obliged to report that a lack of behavioral observations to demonstrate the extent of ‘competition’ renders this result to be treated with some caution. However, larger males have been shown to be better and more intense competitors [13], and are presumed to be of higher quality [19]. It may not be unreasonable to assume that an increasing number of larger males would represent a situation of increased competition (albeit between comparable competitors), and/or female choice (albeit between several, comparable ‘high quality’ males). In any case, what is important to note from these results is that females (exposed to small or large males) appear to have a relatively fixed “arousal threshold” before they accept to copulate with any male, and this threshold is reached faster when females are courted by larger males.

How much of this mating success is driven by male behaviors and how much by female choice? As Partridge et al [13] noted, female rejection behaviors (running away from courting males, dismounting) were not biased towards larger or smaller males. The lack of size-based bias in female rejection behaviors suggests a lack of ‘choice/preference’ behaviors towards male body size during courtship. Rather, we posit that these ‘rejections’ more likely mean that females are not ready to mate yet, although female resistance to male harassment cannot be entirely ruled out. This proposition is logical considering that males are the ones that initiate sexual encounters [1] since they are ready to mate. The same may not be said for females—hence the need for courtship–which is perhaps required to occur for a certain amount of time to elicit physiological/behavioral responses in females to perceive and to become receptive to mate, i.e. the courtship arousal threshold. As such, female ‘acceptance’ behaviors are likely to be influenced to a significant extent by how ‘intensely’ males court females and the signals that they deliver for females’ perception during courtship [3].

The finding that that larger males sing ‘louder’ due to their large thorax and wings [13] may perhaps be extended to other components of the courtship. Signals from all components of *Drosophila* courtship (pursuing, CHC profiles, circling, pulse and sine songs, genital tapping/licking) are perhaps amplified in larger males and are more ‘easily perceptible’ to females. As a result, large males spend less time in each component compared to smaller males who need to spend more time and effort. The mating success of larger males is also a result of larger males being more active during courtship [13,14]. However it is important to note that smaller males also succeed in copulating with females, even in the presence of larger males—they just seem to take longer to court an acceptance response. In the case where two or more males court females at the same time [10] the most active and persistent would gain advantage. As such, we would surmise that the most aggressive/persistent of smaller males will gain mating advantage, as it would be amongst large males. Many of these courtship components are quantifiable (e.g., song in [13]) and a study designed to measure the differences in each component between large and small males will be very useful in testing our proposition. If our interpretations are correct, then the evolution of male traits is influenced to a greater extent by males’ own behavior, particularly heightened activity and pursuits during courtship. However, these results do not necessarily eliminate female choice from mating negotiations because ‘rejection’ (by reverse logic) may also imply the existence of some form of ‘choice/preference’ on the part of females. Moreover, the variation in male activity provides a substrate for female choice.

The distinction between ‘choice’ and ‘acceptance’ behavior of females is clearly made in the copulation interruption assays. On commencement of courtship, once the courtship arousal threshold is reached, a female allows a male to copulate with her. But when copulation is interrupted, the subsequent post-arousal CATs during re-mating are significantly reduced regardless of male size (Table 1, Fig 3). Interestingly, in these assays, mating success after copulation interruption follows the results of Pitnick [19], that larger males secured more copulations in first and second mating. However, the key difference lies in how females respond to males of different sizes once they are ‘sexually aroused’—the females do not mate quicker with a ‘preferred’ male (presumably a larger male as in [19]). Once sexually aroused, there is no difference in the time taken for females to accept large or small males. The higher percentage of copulations (in this study) and re-mating [19] of larger males may therefore be due to their heightened activity, rather than female preference. If indeed there was ‘choice’ involved, the expectation is that females would re-copulate more quickly and more frequently with males that they initially ‘preferred’. This is however not the case in our study, and the result is quite important in demonstrating the parameters of the courtship arousal threshold–that females require a certain level of interaction with the male to become ‘aroused’ or interested in mating. This is quite different from females taking the time to evaluate and choose amongst mate, which does seem to be the case in our study. As a result, our data suggests that a more parsimonious explanation for the mating speed and success of larger males is determined by male behaviors that influence the acceptance response of females. Although these are experimental manipulation, observations in the wild will be quite useful to test the relevance of copulation interruption in the wild. Many factors including wind perturbations, predators or other disturbance are likely to interrupt copulations, particularly within the first few minutes, when genital coupling is not yet complete and easier to disengage copulating pairs [28].

Our results support Darwin’s idea that the activity and effort of males in courtship itself may be under selection, (and in our opinion this is true regardless of an effect of female choice). This idea is recurrent throughout Darwin’s explanations of sexual selection, yet, never emphasized as a potential mechanism by Darwin or subsequent works on sexual selection. Darwin [2] highlighted the importance of male ‘eagerness’ to initiate sexual interactions: ‘*The males*, *as we have seen*, *are generally ready to breed before the females*’(pp261); ‘*the law is*, *that the male shall seek the female*” (pp 272), and as a result ‘*The exertion of some choice on the part of the female seems almost as general a law as the eagerness of the male*” (pp273). He also questioned ‘*why should the male almost always be the seeker*?” (pp273), and later surmised how male ‘*eagerness*’ and ‘*passions*’ to initiate sexual interactions should itself be a trait under strong selection (pp274). Of course, we can expect considerable variation in the relative importance of male behaviors and female ‘choice’ or ‘acceptance’ behaviors across taxa, particularly across taxa with very different mating systems. The variation will also depend on the trait in question. But in many cases, the concept of body size ‘amplifying’ male activity in competition or courtship signals should be relevant. Active female choice may indeed be of higher relative importance in many cases, such as in the case of many birds that compete through displays. However, there is a need to better understand the relevance of displays. For instance, displays may indicate genetic quality either by ‘beauty’ or ‘dominance’ [2,9], or displays and songs may be used for species recognition [29], which may apply particularly to tropical birds who display in thick foliage. Moreover, the same trait such as song or dance may contain overlapping characteristics; for instance in *Drosophila* courtship song, von Schilcher [30] has shown that pulse song may be more relevant to species recognition since song affects female receptivity. As we show in our study, body size may amplify a signal (large males), but the amplification can also be achieved via heightened activity (large or small males).

The extensions of our results to taxa other than *D*. *melanogaster* remain speculations until tested, but our study does bring out the need for revising our understanding of how sexual selection works, and the relative importance of interacting behaviors of the sexes during mating. Testing our results in model organisms where testing female choice is not so tricky (as in *Drosophila*) will be particularly useful. We also urge further research on male sexual behaviors and how they impact female sexual behaviors, (and vice versa) not only with regards to male mate choice [31,32], but also with regards to male persistence and aggression, and its consequences on female responses during mating.

## Supporting Information

S1 Table Influence of male body size on female courtship arousal threshold (CAT); single matings. (XLSX)

S2 Table Influence of male body size on female CAT; ‘competition/choice’ in the presence of a barrier. (XLSX)

S3 Table Influence of male body size on female CAT; ‘competition/choice’ without barriers. (XLSX)

S4 Table Influence of increasing males on male-male competition/female choice. (XLSX)

S5 Table Copulation interruption assays to test for female choice. (XLSX)
